# The full-length nsp2 replicase contributes to viral assembly in highly pathogenic PRRSV-2

**DOI:** 10.1128/jvi.01821-24

**Published:** 2024-11-27

**Authors:** Yuan-Zhe Bai, Shujie Wang, Yue Sun, Yong-Gang Liu, Hong-Liang Zhang, Qian Wang, Rui Huang, Cui-Hong Rao, Shi-Jia Xu, Zhi-Jun Tian, Tong-Qing An, Xue-Hui Cai, Yan-Dong Tang

**Affiliations:** 1 State Key Laboratory for Animal Disease Control and Prevention, Harbin Veterinary Research Institute of Chinese Academy of Agricultural Sciences 687216 , Harbin, Heilongjiang, China; 2 Heilongjiang Provincial Research Center for Veterinary Biomedicine, Harbin Veterinary Research Institute of Chinese Academy of Agricultural Sciences 111613 , Harbin, Heilongjiang, China; 3 Heilongjiang Provincial Key Laboratory of Veterinary Immunology, Harbin Veterinary Research Institute of Chinese Academy of Agricultural Sciences 111613 , Harbin, Heilongjiang, China; Loyola University Chicago - Health Sciences Campus, Maywood, Illinois, USA

**Keywords:** arterivirus, PRRSV, nsp2, assembly, glycoprotein (GP), nucleocapsid (N) protein

## Abstract

Porcine reproductive and respiratory syndrome viruses (PRRSVs) are significant pathogens that affect the global swine industry. Its virions consist of a central core composed of nucleocapsid (N) protein, surrounded by multiple distinct viral envelope proteins. However, the mechanisms underlying the recognition and packaging of N protein by viral envelope proteins remain elusive. In this study, we elucidated the role of nonstructural protein 2 (nsp2) from highly pathogenic PRRSV-2 (HP-PRRSV-2) in viral assembly. Firstly, among all the tested envelope proteins, only glycoprotein 5 (GP5) exhibits limited interaction with N protein. Interestingly, we demonstrated that full-length nsp2 co-immunoprecipitates (Co-IPs) with the N protein and all tested viral envelope proteins. In the presence of full-length nsp2, the N protein interacts with distinct viral envelope proteins. Moreover, upon viral infection, Co-IP experiments using nsp2-specific antibodies or N-specific antibodies revealed the formation of a complex between N and nsp2 with the M protein, GP2a, and GP5. However, neither of the two short forms of nsp2—namely nsp2TF nor nsp2N—participates in this process as they fail to interact with the N protein. Finally, our results demonstrate that this process occurs in the endoplasmic reticulum (ER) and the ER-Golgi intermediate compartment (ERGIC). Overall, our findings unveil a novel functional role for full-length nsp2 of HP-PRRSV-2 in facilitating the assembly of the N protein with viral envelope proteins.

**IMPORTANCE:**

The virus assembly process of arteriviruses remains largely elusive, including the direct interaction between N protein and viral envelope proteins or the potential requirement for additional proteins in facilitating assembly. Moreover, where the N protein assembles with viral envelope proteins during the virus lifecycle remains unclear. This study reveals a novel role for nonstructural protein 2 (nsp2) in highly pathogenic porcine reproductive and respiratory syndrome virus type 2 (HP-PRRSV-2), highlighting its involvement in HP-PRRSV-2 assembly. These findings provide crucial insights into HP-PRRSV-2 assembly and enhance our understanding of their lifecycle. Overall, this study offers an alternative approach to developing a new antiviral strategy targeting PRRSV-2 assembly.

## INTRODUCTION

Arteriviruses belong to the order *Nidovirales* and share some similarities with coronaviruses in genomic organization (1). Arteriviruses are a group of positive-stranded RNA viruses that primarily infect mammals. This viral family includes equine arteritis virus, mice lactate dehydrogenase-elevating virus, porcine reproductive and respiratory syndrome virus type 1 and porcine reproductive and respiratory syndrome virus type 2 (PRRSV-1 and PRRSV-2), simian hemorrhagic fever virus, as well as wobbly possum disease virus (2–4). These viruses can result in infected animals exhibiting persistent or asymptomatic infections, along with acute respiratory syndrome, abortion, or even fatal hemorrhagic fever (2, 3). The global pig industry has suffered significant losses primarily due to the devastating impact of PRRSV, making it the most renowned among all arteriviruses (5). In particular, in 2006, China experienced an outbreak of highly pathogenic PRRSV-2 (HP-PRRSV-2), which caused a large number of pig deaths and resulted in huge losses for pig farms (6–8). The genomic RNA of PRRSV is approximately 15 kb in length and encodes the RNA replicase (ORF1a and ORF1b), as well as major envelope proteins including glycoproteins 5 (GP5) and non-glycosylated M protein, along with minor envelope proteins such as GP2a, GP3, GP4, a small E protein, and ORF5a (5). The viral particles consist of an internal nucleocapsid core and outer viral envelope proteins. PRRSV genomic RNA is initially encapsulated by the nucleocapsid (N protein), followed by the formation of disulfide-linked homodimers by the N protein during virus particle budding (9–11). Additionally, the N-terminal domain exhibits a high abundance of positively charged residues, potentially facilitating its interaction with viral RNA (10). According to cryo-electron microscopy studies of PRRSV particles, the viral core has an average diameter of 39 nm, which is thought to be formed by two layers of N protein dimers. The viral core is separated from the envelope by a 2–3 nm gap (12). The gap between the core and the envelope, traversed only by a few strands of density, indicates that the interactions between the nucleocapsid core and its surface envelope proteins, if present, are weak and flexible (12).

PRRSV envelope is composed of major envelope proteins (GP5 and M protein) and minor envelope proteins (GP2a, GP3, GP4, E protein, ORF5a). GP5 and M protein form a disulfide-linked heterodimer, which is essential for arterivirus assembly (13–15). The formation of arterivirus particles requires both major envelope proteins; if either GP5 or the M protein is absent, no particles will be produced (16). The minor envelope proteins, GP2a, GP3, and GP4, form a heterotrimer on the virion envelope (17, 18). All these envelope proteins are essential for successful PRRSV infection. For example, the M protein is thought to interact with heparin sulfate and GP5 is recognized to interact with sialoadhesin to aid PRRSV attachment (18–20). GP2a or GP4 is reported to cooperate with the PRRSV key receptor, CD163, to complete the internalization of the virion, uncoating, and the release of the virus nucleic acid (21–23). Interestingly, Kappes et al. showed that nonstructural protein 2 (nsp2) is also present on or within the virions (24). Different isoforms of nsp2 have been identified in PRRSV, including the full-length nsp2, nsp2TF, and nsp2N (25). The nsp2TF is expressed through a −2 programmed ribosomal frameshift (PRF) mechanism (26). Nsp2N is expressed through a −1 PRF that encounters an immediate stop codon, resulting in the production of a truncated nsp2 protein (27, 28). In a recent study, Guo et al. revealed that nsp2TF interacts with the GP5 and M proteins, thereby facilitating the assembly and budding process of PRRSV (29).

Virus assembly is tightly orchestrated during the virus lifecycle (30). However, the assembly mechanism of PRRSV remains largely elusive, particularly with regard to the intricate recognition process between the N protein and various viral membrane proteins. Furthermore, where N protein interacts with viral envelope proteins during assembly is still unclear. Herein, we have discovered that full-length nsp2 exhibits robust interactions with N protein and all tested envelope proteins of HP-PRRSV-2. Furthermore, we have demonstrated that the assembly of N protein with distinct envelope proteins involves full-length nsp2 rather than its truncated forms (nsp2TF or nsp2N). Additionally, our findings reveal that the interaction between N protein and envelope proteins primarily occurs in the endoplasmic reticulum (ER) and ER-Golgi intermediate compartment (ERGIC). Therefore, this study uncovers a novel role for full-length nsp2 of HP-PRRSV-2.

## RESULTS

### The interaction between PRRSV-2 N protein and different viral envelope proteins

**Fig 1:**
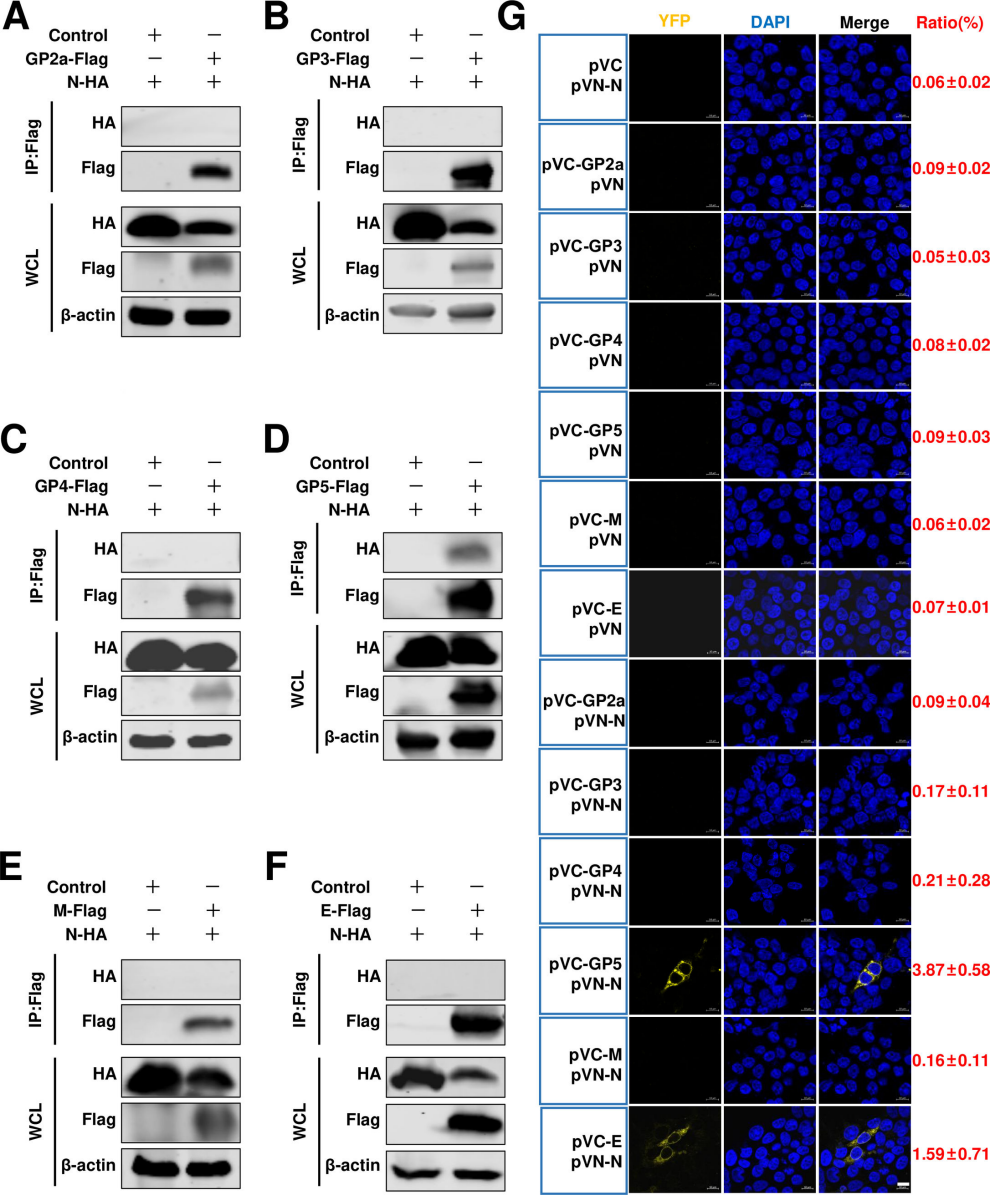
Investigating the interaction between the N protein and envelope proteins. (**A**) HEK293T cells were cotransfected pCAGGS-N-HA with pCAGGS-GP2a-Flag, or (**B**) pCAGGS-GP3-Flag, or (**C**) pCAGGS-GP4-Flag, or (**D**) pCAGGS-GP5-Flag, or (**E**) pCAGGS-M-Flag, or (**F**) pCAGGS-E-Flag. HEK293T cells were cotransfected pCAGGS-N-HA with pCAGGS vector as a control. At 24 hpt, the cells were lysed for Co-IP assay by Flag beads. (**G**) BiFC assay of the N protein and indicated envelope proteins. The nucleus was stained with DAPI (blue). The yellow fluorescence signals (yellow) were observed using the LSM 980 Zeiss confocal microscope. Scale bars, 10 µm. The YFP fluorescence signals were further detected via flow cytometry, and the ratio of YFP fluorescence-positive cells was listed on the right. The experiments were independently repeated three times, and representative data are shown.

**Fig 2:**
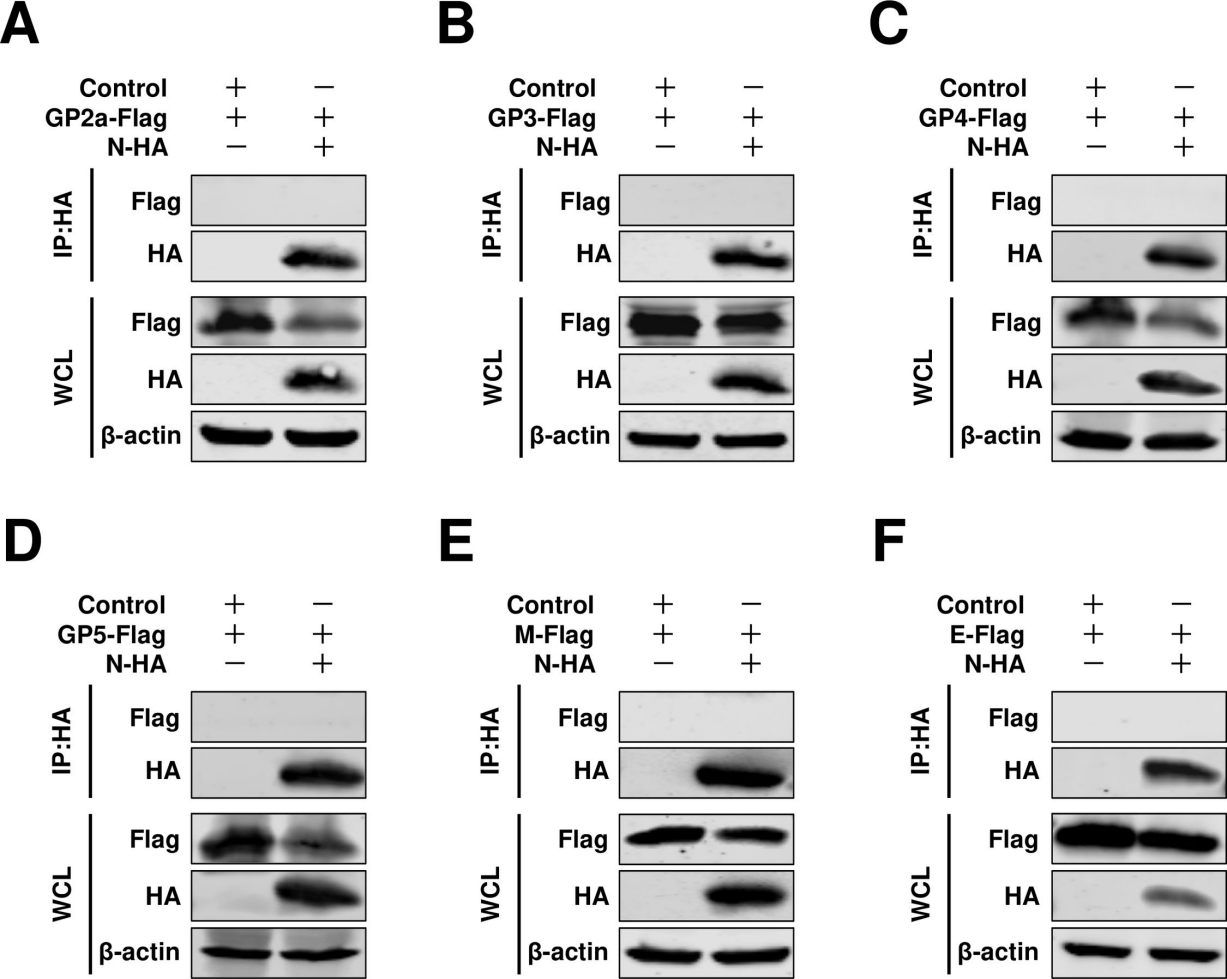
Co-IP experiments were conducted using N protein. (**A**) HEK293T cells were cotransfected pCAGGS-GP2a-Flag, or (**B**) pCAGGS-GP3-Flag, or (**C**) pCAGGS-GP4-Flag, or (**D**) pCAGGS-GP5-Flag, or (**E**) pCAGGS-M-Flag, or (**F**) pCAGGS-E-Flag with pCAGGS-N-HA, and with pCAGGS vector as a control. At 24 hpt, the cells were lysed for Co-IP assay by HA beads. The experiments were independently repeated three times, and representative data are shown.

Our hypothesis was that if viral nucleocapsid cores are indeed packaged by these envelope proteins, the initial interaction should occur between the N protein and these specific viral envelope proteins. To investigate this, we utilized the HP-PRRSV-2 HuN4 strain and constructed expression plasmids for Flag-tagged envelope proteins and HA-tagged N proteins. When we conducted a co-immunoprecipitate (Co-IP) assay with Flag beads, we found there was no detectable interaction between N protein and GP2a, GP3, GP4, M, or E proteins except GP5 at 24 hours post-transfection (hpt) (Fig. 1A through F). We next further confirmed these by a Co-IP assay with N protein, therefore, we used HA beads, to our surprise, no envelope proteins were IP-ed with N protein (Fig. 2). Subsequently, we further validated these findings using a bimolecular fluorescence complementation (BiFC) assay, which enables direct visualization of protein interactions in live cells (31–33). Notably, only the pVN-N/pVC-GP5 and pVN-N/pVC-E exhibited limited detectable fluorescence signals (Fig. 1G). We next quantified the number of cells that were fluorescence-positive in transfected cells using a flow cytometer. The results revealed that only 3.87% ± 0.58% and 1.59% ± 0.71% of cells exhibited fluorescence positivity for pVN-N/pVC-GP5 and pVN-N/pVC-E, respectively (Fig. 1G; Fig. S1). This observation suggests that if any interactions exist, they may be weak and insufficient for viral particle assembly.

### The nsp2 is associated with the N protein and viral envelopes

**Fig 3:**
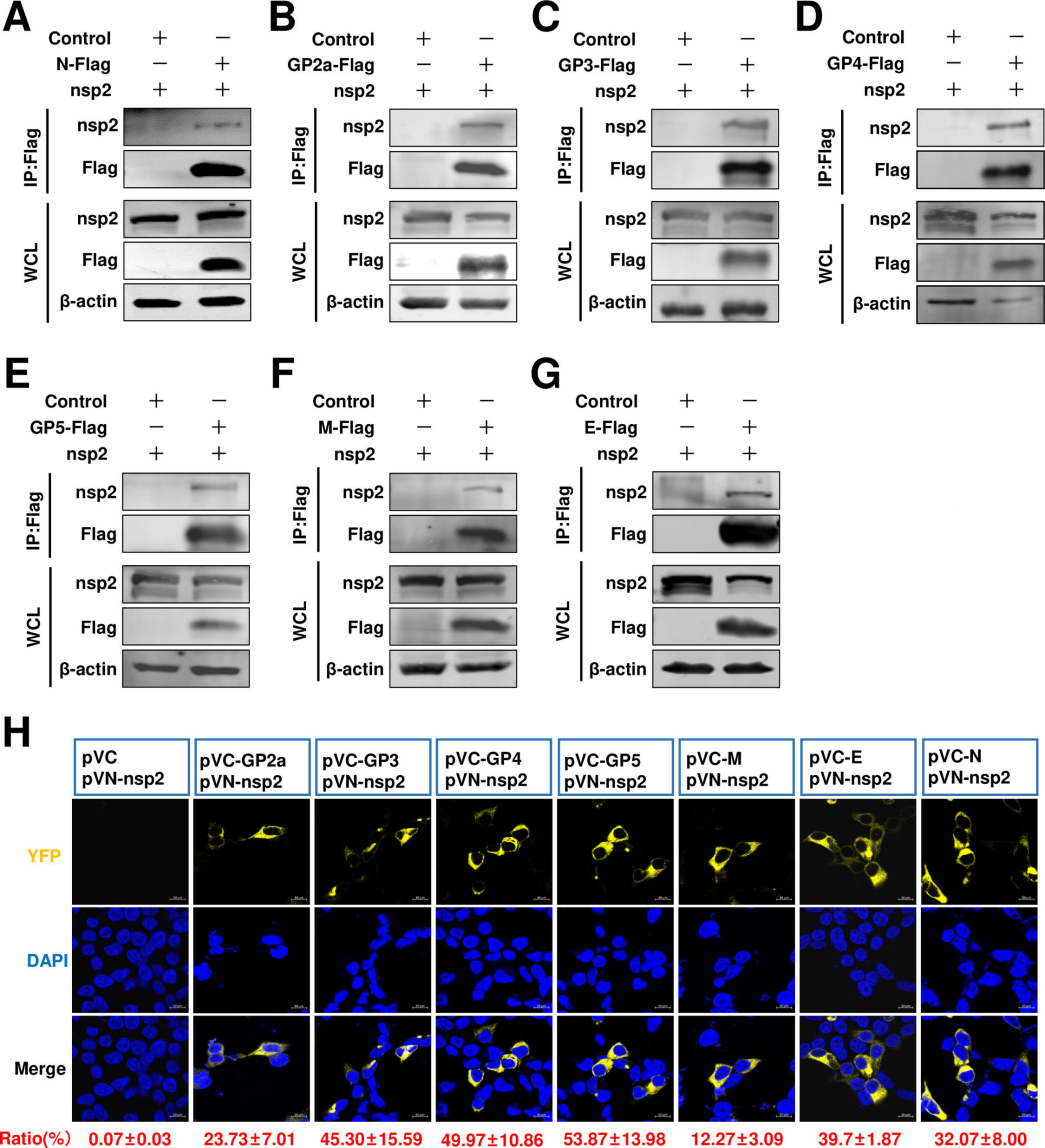
Nsp2 interacts with both the N protein and the viral envelopes. (**A**) HEK293T cells were cotransfected pCAGGS-nsp2 with pCAGGS-N-Flag, or (**B**) pCAGGS-GP2a-Flag, or (**C**) pCAGGS-GP3-Flag, or (**D**) pCAGGS-GP4-Flag, or (**E**) pCAGGS-GP5-Flag, or (**F**) pCAGGS-M-Flag, or (**G**) pCAGGS-E-Flag. Cells were cotransfected pCAGGS-nsp2 with pCAGGS vector as a control. At 24 hpt, the cells were lysed for Co-IP assay. The Co-IP samples were then analyzed by western blot. (**H**) The nsp2 and indicated envelope proteins were analyzed using the BiFC assay. HEK293T cells were cotransfected with pVN-nsp2 and pVC-GP2a, pVC-GP3, pVC-GP4, pVC-GP5, pVC-M, pVC-E, pVC-N, or pVC. At 24 hpt, the cells were fixed, permeabilized, and stained with DAPI (blue). YFP fluorescence signals were detected using a confocal microscope and quantified using flow cytometry. The experiments were repeated independently three times and representative data are shown.

**Fig 4:**
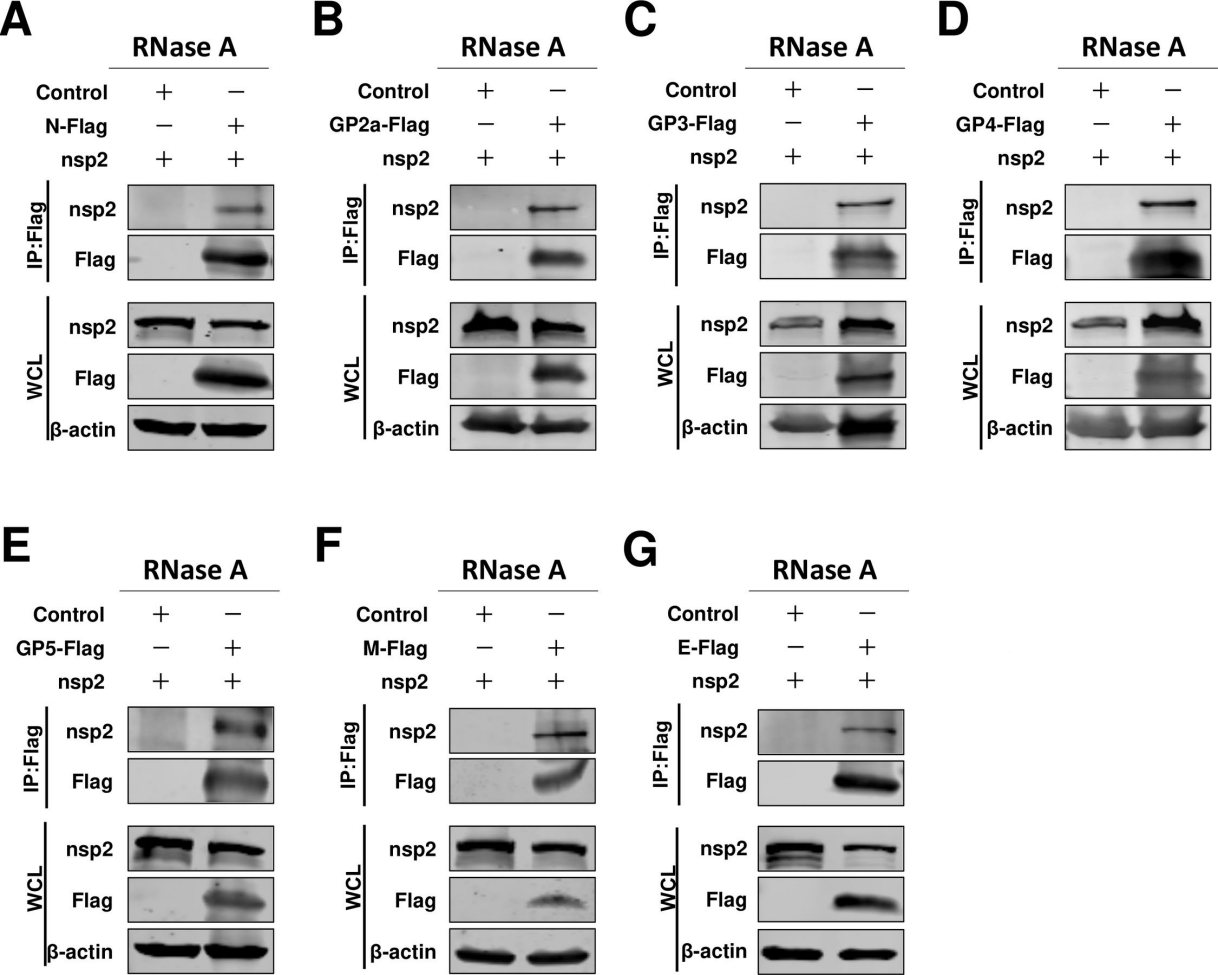
Nsp2’s interaction with viral structural proteins was not mediated by RNA. (**A**) HEK293T cells were cotransfected pCAGGS-nsp2 with pCAGGS-N-Flag, or (**B**) pCAGGS-GP2a-Flag, or (**C**) pCAGGS-GP3-Flag, or (**D**) pCAGGS-GP4-Flag, or (**E**) pCAGGS-GP5-Flag, or (**F**) pCAGGS-M-Flag, or (**G**) pCAGGS-E-Flag. Cells were cotransfected pCAGGS-nsp2 with pCAGGS vector as a control. The cells were lysed and treated with RNase A at 24 hpt, followed by subsequent Co-IP assay. Subsequently, the Co-IP samples underwent western blot analysis. The experiments were independently repeated three times, and representative data are presented.

We next aimed to elucidate the incorporation mechanisms of other viral envelope proteins, particularly GP2a, GP3, and GP4, into viral particles. We hypothesized that other viral proteins may facilitate assembly between N protein and envelope proteins. This putative viral protein bridged connecting the N protein and viral envelope proteins was likely to be a transmembrane or structural protein. In a previous investigation, the nsp2 replicase protein was confirmed as a viral transmembrane structural component and subsequently incorporated into virions (24). Therefore, we tested whether nsp2 was involved in viral assembly. Interestingly, through a Co-IP assay, we found that nsp2 IPed with N protein (Fig. 3A). After confirming that nsp2 interacted with N protein, we next evaluated whether nsp2 also interacted with other viral envelope proteins. Our results indicated that all of these viral envelope proteins could be IPed with nsp2 (Fig. 3B through G). The role of RNA in mediating protein-protein interactions is well-established. To investigate the potential involvement of RNA in the interaction between nsp2 and other viral proteins, we employed RNase A treatment on HEK293T cell samples to assess these interactions. Remarkably, our findings demonstrated that the interaction persists even in the presence of RNase A (Fig. 4). To further confirm and visualize these potential interactions, we also performed a BiFC assay. We found that fluorescence signals could be detected in all tested groups, except for the control group (Fig. 3H). We quantified the rate of positive fluorescence cells and found that in pVC-N/pVN-nsp2 transfected cells, the ratio of fluorescence-positive cells was 32.07% ± 8.00% (Fig. 3H). The ratio of fluorescence-positive cells in the pVN-nsp2 and pVC-GPs groups ranged from 12.27% ± 3.09% to 53.87% ± 13.98% (Fig. 3H; Fig. S2). These fluorescence-positive cells were significantly higher than those in the pVN-N/pVC-GP5 and pVN-N/pVC-E groups. Overall, we demonstrated that nsp2 interacted with N protein and all of the tested viral envelope proteins. Therefore, we recognized that nsp2 may play a critical role in HP-PRRSV-2 assembly.

### Nsp2 promotes the assembly of the N protein with the viral envelope proteins

**Fig 5:**
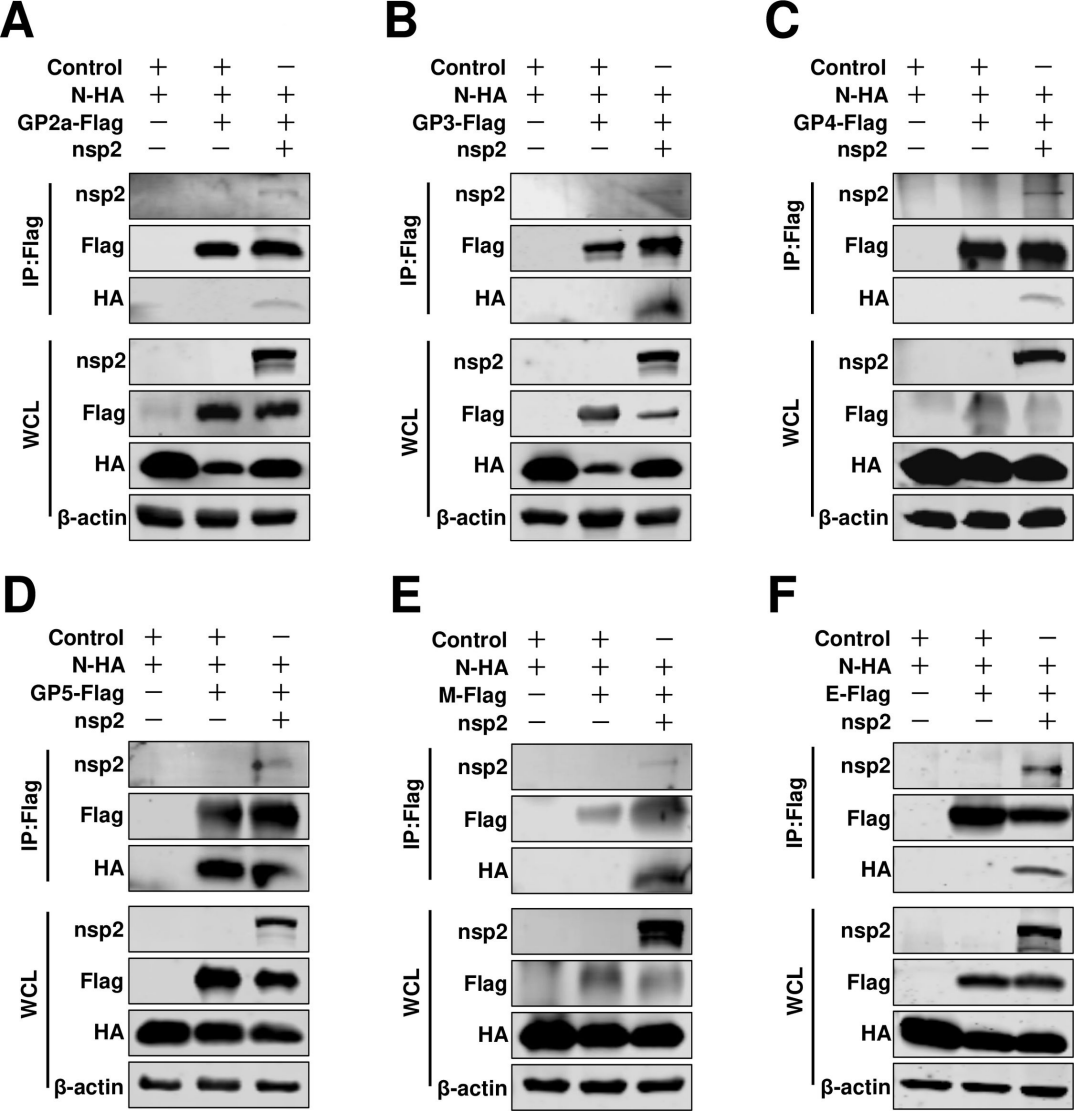
Nsp2 promotes the assembly of N protein with viral envelope proteins. (**A**) HEK293T cells were cotransfected pCAGGS-nsp2 with pCAGGS-N-HA with pCAGGS-GP2a-Flag, or (**B**) pCAGGS-GP3-Flag, or (**C**) pCAGGS-GP4-Flag, or (**D**) pCAGGS-GP5-Flag, or (**E**) pCAGGS-M-Flag, or (**F**) pCAGGS-E-Flag, or control plasmids. At 24 hpt, the cells were lysed for Co-IP assay. The Co-IP samples were then analyzed by western blot.

**Fig 6:**
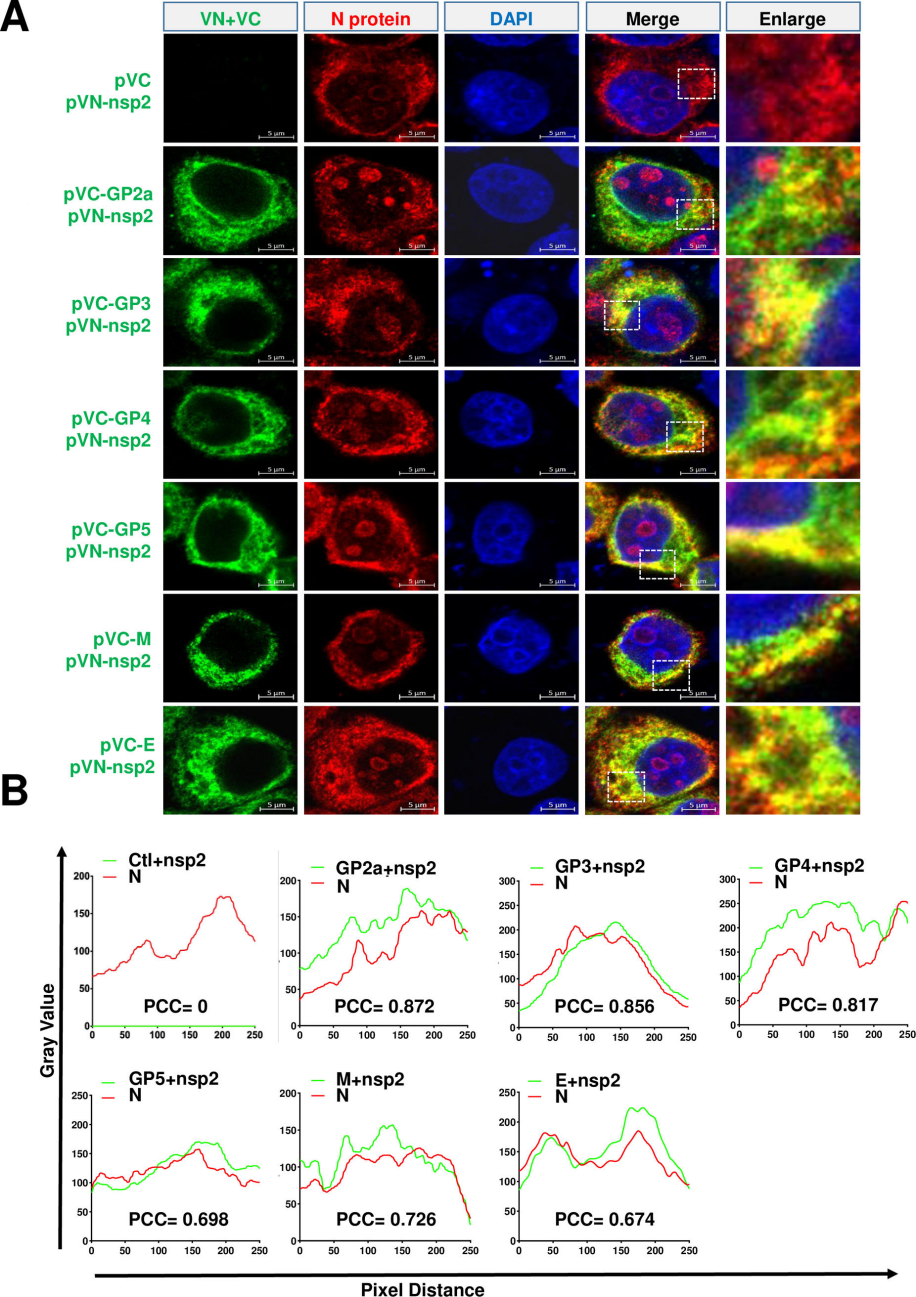
Co-localization analysis of N protein, nsp2, and viral envelope proteins. (**A**) HeLa cells were co-transfected with pCAGGS-N-Flag and pVN-nsp2 along with either pVC-GP2a, pVC-GP3, pVC-GP4, pVC-GP5, pVC-M, pVC-E, or control plasmids. After 24 hpt, the cells were fixed, permeabilized, and subjected to immunostaining for N protein (red). Fluorescent signals were visualized using the LSM 980 Zeiss confocal microscope. Scale bars of 5 µm are shown in the images. Representative co-localization images (yellow) are presented. The experiments were independently repeated three times and representative data is displayed. (**B**) PCC was utilized to quantify the level of co-localization between nsp2 and N protein with GP2a, GP3, GP4, GP5, M, or E protein using Image J software.

Next, we attempted to test whether nsp2 was involved in the assembly of the N protein with envelope proteins. Interestingly, in the presence of nsp2, these minor envelope proteins were able to IP with N protein (Fig. 5A through C). We further evaluated the major envelope protein GP5 and found that, regardless of the presence of nsp2, it could IP with the N protein (Fig. 1D and 5D). For the M and E proteins, it could only IP with the N protein in the presence of nsp2 (Fig. 5E and F). We further performed a confocal assay to determine the co-localization of the N protein, nsp2, and viral envelope proteins. We used pVN-nsp2 and pVC-GPs, pVC-M, or pVC-E to visualize the interaction of nsp2 and envelope proteins (GP2a, GP3, GP4, GP5, M, and E), and further visualized the N protein using its specific antibody. Our results indicated that the N protein, nsp2, and all tested viral envelope proteins exhibited strong co-localization (Fig. 6A). The level of co-localization was quantified by calculating Pearson’s correlation coefficient (PCC) using ImageJ software. The results further showed that the N protein, nsp2, and viral envelope proteins were highly co-localized (Fig. 6B). Therefore, we concluded that nsp2 was a key mediator in promoting the assembly of the N protein with viral envelope proteins.

### The nsp2 forms a complex with N, M, GP2a, and GP5 during viral infection

**Fig 7:**
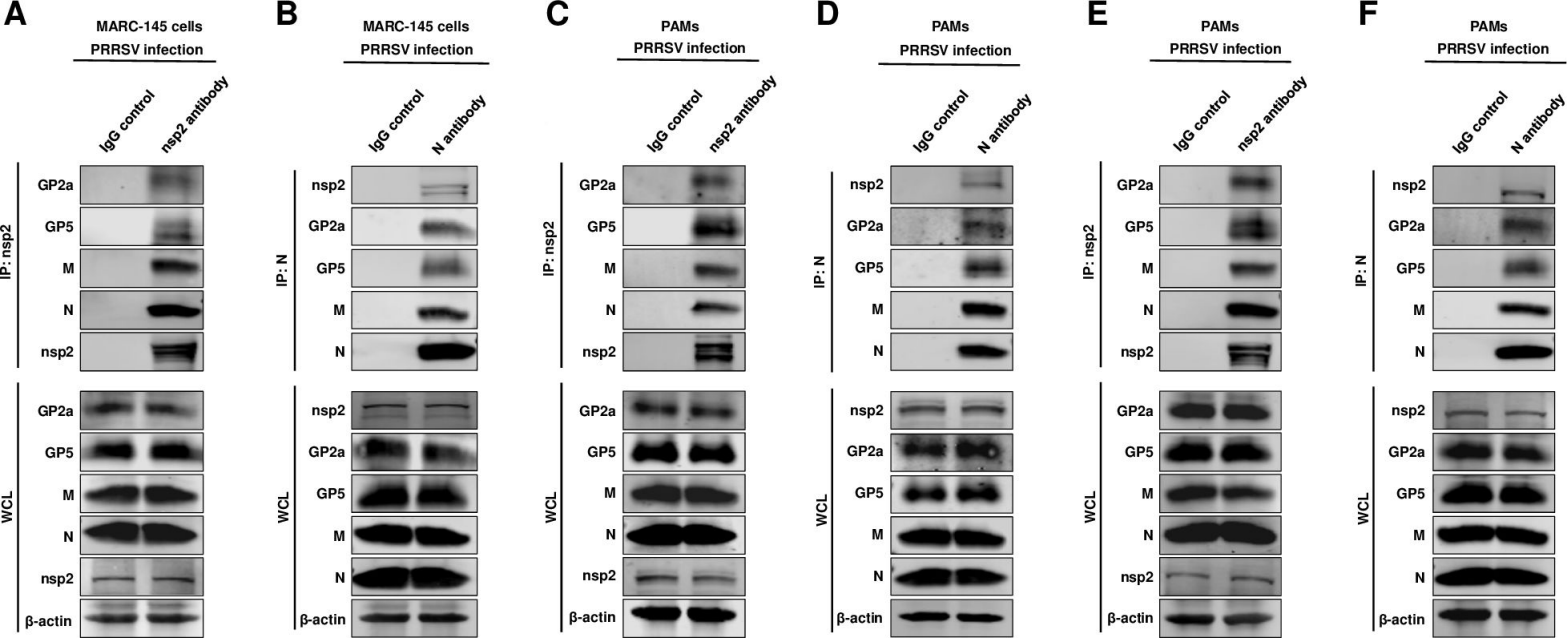
The nsp2 forms a complex with N, M, GP2a, and GP5 during viral infection. (**A**) The MARC-145 cells were infected with PRRSV HuN4 at an MOI of 0.1. After 48 hours post-infection, the cell lysates were collected and subjected to Co-IP assay using anti-nsp2 monoclonal antibody (MAb) or (**B**) using anti-N protein monoclonal antibody. Additionally, IgG was used for immunoprecipitation as a control. (**C**) The PAMs were infected with PRRSV HuN4 at an MOI of 0.1. After 24 hours post-infection, the cell lysates were collected and subjected to Co-IP assay using an anti-nsp2 antibody or (**D**) using an anti-N protein monoclonal antibody. IgG was used for immunoprecipitation as a control. (**E**) The PAMs were infected with HP-PRRSV-like strain at an MOI of 0.1. After 24 hours post-infection, the cell lysates were collected and subjected to Co-IP assay using an anti-nsp2 antibody or (**F**) using an anti-N protein monoclonal antibody. IgG was used for immunoprecipitation as a control.

We next investigated the interaction between nsp2 and viral structural proteins during PRRSV infection. If nsp2 is involved in the assembly of N protein and envelope proteins during viral replication, it is expected to form a complex with both the N protein and distinct viral envelope proteins. Therefore, we infected MARC-145 cells with the HP-PRRSV HuN4 strain at an MOI of 0.1. After 48 hours, lysates from the infected cells were subjected to an IP assay using an nsp2 antibody. Our findings demonstrated that nsp2 interacts with GP2a, GP5, N protein, and M protein (Fig. 7A). To further confirm the formation of this complex, we next performed an IP assay using the N antibody and found that nsp2, GP2a, GP5, and M were all IPed with the N protein (Fig. 7B). We also conducted these experiments in porcine alveolar macrophages (PAMs), which are target cells for PRRSV infection *in vivo*, and observed the formation of the complex (Fig. 7C and D). Furthermore, we tested another field-isolated HP-PPRSV-like strain which is similar to HP-PRRSV, and the results confirmed the formation of this complex as well (Fig. 7E and F). These results provide support for nsp2’s role as a mediator facilitating assembly between viral envelope proteins and N protein during HP-PRRSV infection.

### The interaction between nsp2 truncation isoforms (nsp2N and nsp2TF) and structural proteins

**Fig 8:**
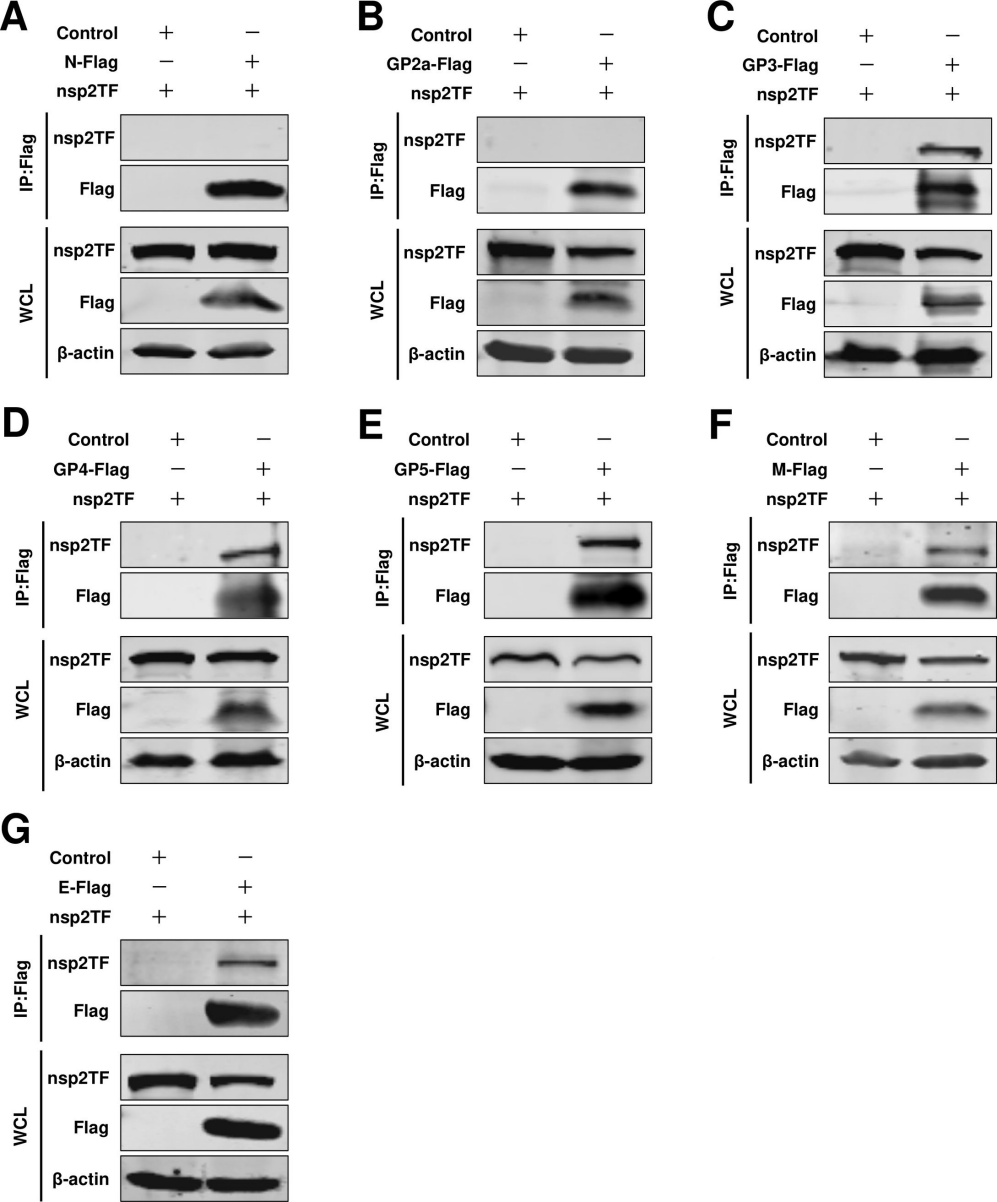
The interaction between nsp2TF and structural proteins. (**A**) HEK293T cells were cotransfected pCAGGS-nsp2TF with pCAGGS-N-Flag, or (**B**) pCAGGS-GP2a-Flag, or (**C**) pCAGGS-GP3-Flag, or (**D**) pCAGGS-GP4-Flag, or (**E**) pCAGGS-GP5-Flag, or (**F**) pCAGGS-M-Flag, or (**G**) pCAGGS-E-Flag. Cells were cotransfected pCAGGS-nsp2TF with pCAGGS empty vector as a control. At 24 hpt, the cells were lysed for Co-IP assay. The Co-IP samples were then analyzed by western blot.

**Fig 9:**
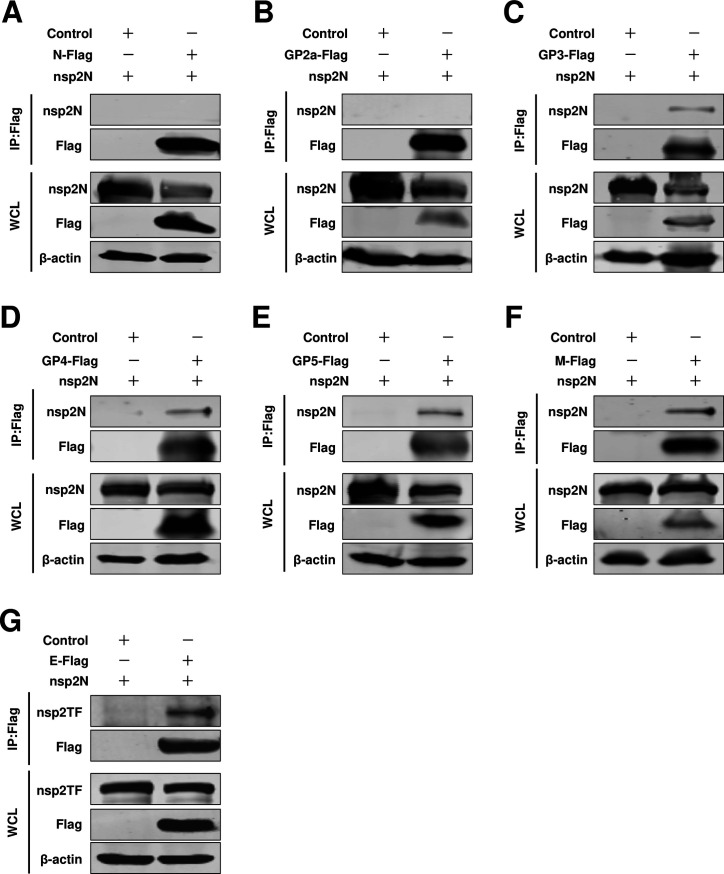
The interaction between nsp2N and structural proteins. (**A**) HEK293T cells were cotransfected pCAGGS-nsp2N with pCAGGS-N-Flag, or (**B**) pCAGGS-GP2a-Flag, or (**C**) pCAGGS-GP3-Flag, or (**D**) pCAGGS-GP4-Flag, or (**E**) pCAGGS-GP5-Flag, or (**F**) pCAGGS-M-Flag, or (**G**) pCAGGS-E-Flag. Cells were cotransfected pCAGGS-nsp2N with pCAGGS empty vector as a control. At 24 hpt, the cells were lysed for Co-IP assay. The Co-IP samples were then analyzed by western blot.

In addition to the full-length nsp2, there are two distinct truncation isoforms of nsp2, namely nsp2TF and nsp2N (26, 27). Compared to the full-length nsp2, they exhibit distinct C-terminal regions. nsp2TF plays a crucial role in PRRSV assembly by interacting with GP5 and M (29). Therefore, we investigated whether these truncation isoforms exhibit identical functionality when the N protein assembles with envelope proteins. Through a Co-IP assay, we observed that the viral envelope proteins GP3, GP4, GP5, E, and M exhibited persistent interactions with nsp2TF (Fig. 8C through F). Conversely, N protein and GP2a failed to interact with nsp2TF (Fig. 8A and B). We obtained similar results for nsp2N (Fig. 9). These findings suggested that neither nsp2TF nor nsp2N exhibited interactions with the N protein, thus failing to serve as a protein bridge for facilitating assembly between N protein and viral envelope proteins. Therefore, we proposed that the full-length nsp2, rather than nsp2N and nsp2TF, acted as a mediator for the assembly of N protein with envelope proteins.

### The assembly of N protein with envelope proteins occurs in ER and ERGIC

**Fig 10:**
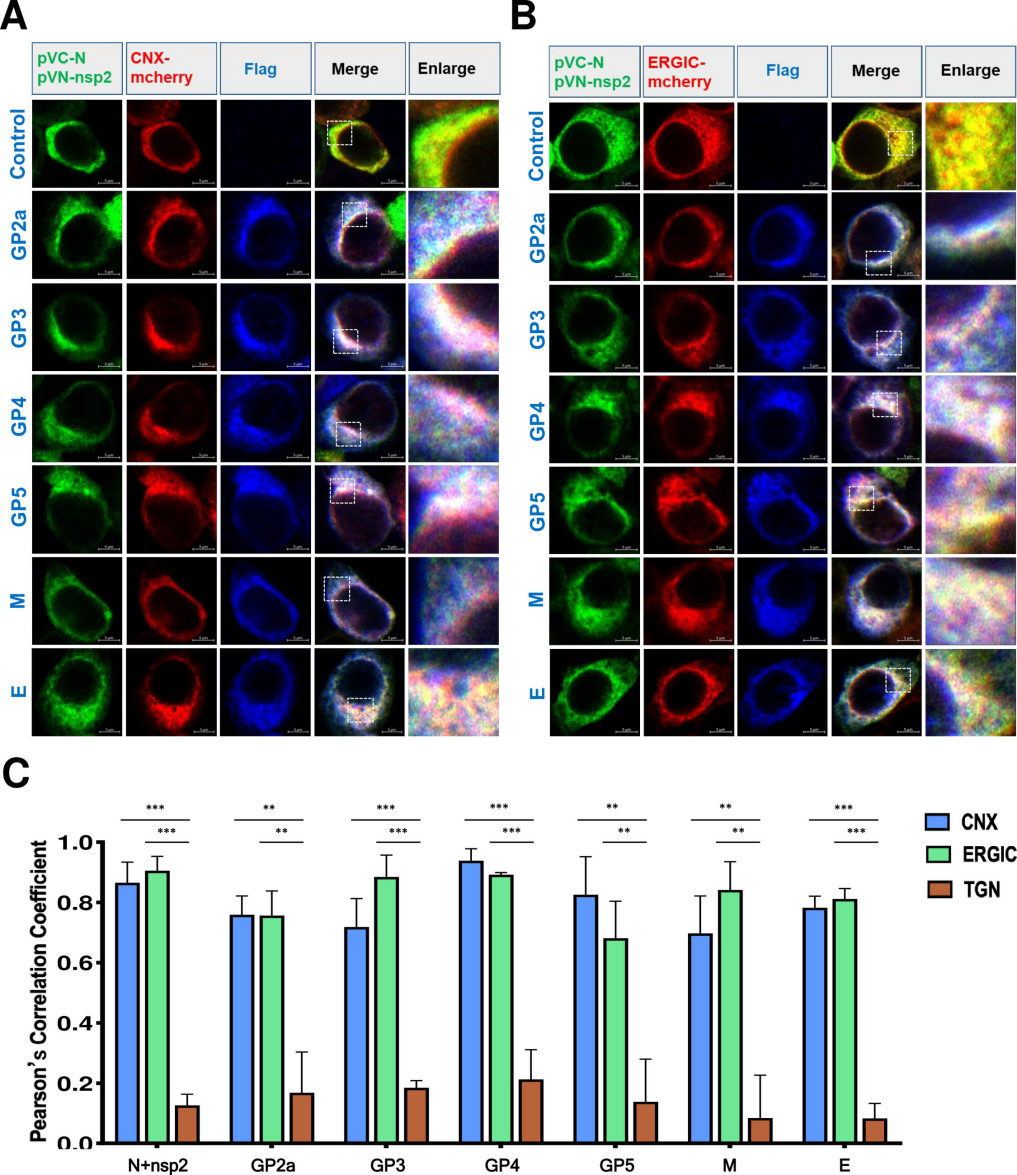
The assembly mainly occurs in ER and ERGIC. (**A**) The HeLa cells were co-transfected with pVC-N and pVN-nsp2, an indicated plasmid expressing viral envelope protein, along with either CNX-mCherry or (**B**) ERGIC-53-mCherry as markers. After 24 hours of transfection, the cells were fixed, permeabilized, and subjected to immunostaining. Scale bars representing 5 µm were included in the images. The experiments were independently repeated three times, and representative data are presented. (**C**) The level of co-localization between CNX, ERGIC, or TGN and (N+nsp2), GP2a, GP3, GP4, GP5, M, or E protein was quantified by PCC using ImageJ software. The mean (±SD) values from three replicates are shown. **P < 0.01; ***P < 0.001; Student’s t-test.

Next, we aimed to determine which intracellular organelles supported this assembly. The ER was labeled with Calnexin-mCherry (CNX-mCherry), the ERGIC was labeled with ERGIC-53-mCherry (ERGIC-mCherry), and the trans-Golgi network (TGN) was labeled with TGN46-mCherry (TGN-mCherry), respectively. Viral envelope proteins were labeled with a Flag tag antibody and the interaction between nsp2 and N was observed by BiFC assay. A high degree of co-localization between nsp2, N, and the envelope proteins with the ER and ERGIC was illustrated (Fig. 10A and B). However, for the TGN, very few co-localizations were observed (Fig. S3). We concluded this assembly may occur at ER and ERGIC rather than TGN. The level of co-localization was quantified by calculating the PCC using ImageJ software. The results further showed that nsp2, N, and envelope proteins were significantly co-localized with the ER and ERGIC, but not with the TGN (Fig. 10C).

**Fig 11:**
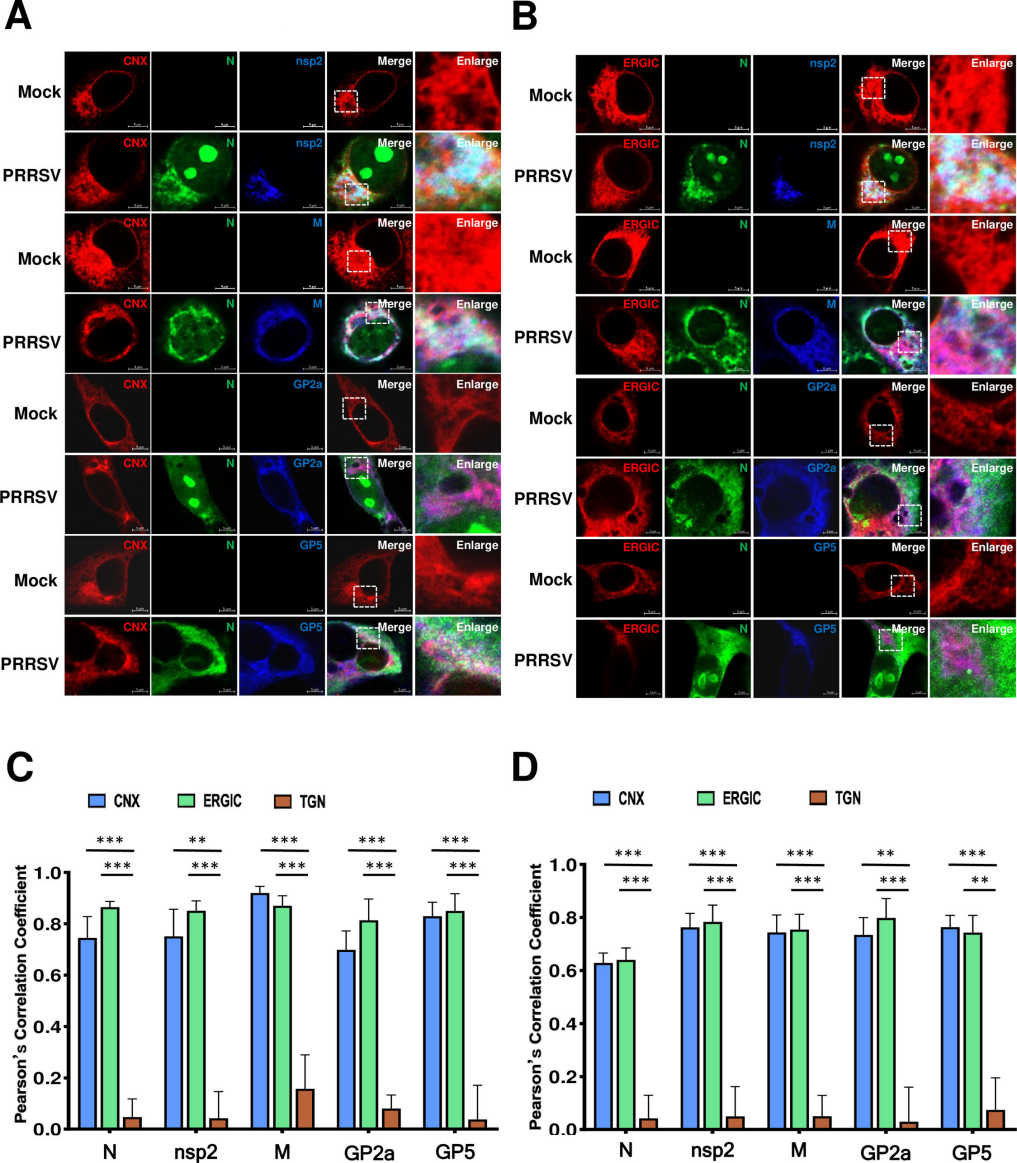
PRRSV infection assembly sites. (**A**) MARC-145 cells were transfected with CNX-mCherry or (**B**) ERGIC-53-mCherry. At 24 hpt, the cells were infected with PRRSV HuN4 (0.1 MOI), with uninfected cells as control. At 48 hours post-infection, the cells were fixed, permeabilized, immunostained for PRRSV nsp2, or GP2a, or GP5, or M protein (blue), and re-immunostained with FITC-conjugated PRRSV/NP Mab (green). Scale bars: 5 µm. The experiments were repeated independently three times, and representative data are shown. (**C**) Co-localization levels between CNX, ERGIC, or TGN and indicated viral proteins were quantified by PCC using ImageJ software. (**D**) MARC-145 cells were transfected with CNX-mCherry, ERGIC-53-mCherry, or TGN-mCherry and infected with HP-PRRSV-like virus (0.1 MOI) as described. The co-localization level of CNX, ERGIC, or TGN with the indicated viral proteins was quantified by PCC via Image J software. Mean (±SD) values from three replicates are presented. **P < 0.01; ***P < 0.001; Student’s t-test.

To further confirm the ER and ERGIC support the assembly of PRRSV during viral infection, we attempted to visualize the intracellular localization of viral proteins. MARC-145 cells were transfected with organelle indicator proteins CNX-mCherry, ERGIC-53-mCherry, or TGN-mCherry. Twenty-four hours post-transfection, the cells were infected with the PRRSV HuN4 strain. Due to the availability of PRRSV antibodies, we only visualized the intracellular organelle localization of the N protein, nsp2, M protein, GP2a, and GP5. We found that the nsp2, N protein, M protein, GP2a, and GP5 showed a high degree of co-localization with the ER and ERGIC (Fig. 11A and B), while the TGN did not (Fig. S4). The analysis of PCC for co-localization was also performed, and the results showed that the viral nsp2, N protein, M protein, GP2a, and GP5 have high PCC values with the ER and ERGIC, but not the TGN (Fig. 11C). Last, we further confirm this used a field-isolated HP-PPRSV-like strain (Fig. 11D). These results further supported that the ER and ERGIC were the main assembly sites for N protein and viral envelope proteins.

## DISCUSSION

In the present study, for HP-PRRSV, we identified a novel role of full-length nsp2 in facilitating the assembly of N protein and viral envelope proteins. Although an interaction between GP5 and the N protein was observed, it was only detected in a limited number of cells in a BiFC assay (Fig. 1G). Moreover, when we conducted a co-IP assay with N antibodies, their interaction was not detected (Fig. 2D). A previous study conducted by Lee et al. indicated the interaction between the E and N proteins through a pulldown assay (34). Interestingly, in their study, this interaction was not detected using a co-IP assay. In our current investigation, we also failed to detect this interaction when performing a co-IP assay. To further validate these potential interactions, we employed a BiFC assay and only observed minimal signals of interaction between the E protein and N protein (Fig. 1G). However, in the presence of nsp2, the interaction between the E protein and N protein became readily detectable (Fig. 5F), suggesting that nsp2 mediates this interaction effectively. These findings imply that if there is indeed an association between GP5 or E with N protein, it may be relatively weak and insufficient for HP-PRRSV assembly since other viral envelope proteins are also required for virion incorporation. In fact, nsp2 is a multifunctional protein in the lifecycle of PRRSV. It plays a key role in the formation of the viral replication complex, evasion of the innate immune response, determination of cell tropism, and modulation of viral virulence and persistence (25, 28, 35–39). PRRSV nsp2 contains a cysteine protease domain in its N-terminal region, which displays both cis- and trans-cleavage activities (25, 40). Comparisons of distinct PRRSV isolates indicated that nsp2 has a hypervariable region (41–43). Furthermore, nsp2 has three different isoforms, including the full-length nsp2, nsp2TF, and nsp2N (25). All three forms of PRRSV nsp2 have been reported to be essential for suppressing the immune responses of the host (39, 44–47). A recent study indicated that nsp2TF interacts with the viral GP5 and M protein, which facilitates arterivirus assembly by counteracting the ubiquitination-dependent proteasomal degradation of GP5 and M protein (29). In our study, we observed that the interaction between nsp2TF/nsp2N and structural proteins, including GP3, GP4, GP5, E, and M, was evident; however, no interaction was detected with GP2a by IP assay. We speculated that nsp2TF and nsp2N do not appear to contribute to the assembly between the N protein and viral envelopes, as no detectable interaction was detected between nsp2TF/nsp2N and the N protein (Fig. 8A and 9A). The full-length nsp2 interacted with all of the tested viral envelopes and N protein. This suggests that the full-length nsp2 may play a more important role in HP-PPRSV assembly than nsp2TF and nsp2N.

Coronaviruses, as members of the *Nidovirales*, have structural proteins that include the hemagglutinin esterase (HE), the spike (S) glycoprotein, the membrane (M) glycoprotein, and the nucleocapsid (N) protein. The M protein plays a fundamental role in coronavirus assembly, as it interacts with all of the viral envelope proteins and the N protein, directing the assembly of the virion (48–51). Unlike coronaviruses, in this study, the M protein of HP-PRRSV did not interact directly with the N protein in the absence of nsp2, suggesting that, although coronaviruses and arteriviruses are all grouped into the order *Nidovirales*, they may have distinct assembly mechanisms. The nsp2 appears to be crucial for porcine arterivirus assembly, as it interacts with all of the tested viral structural proteins. In most coronaviruses, M proteins form homodimers (52), which appear to be functionally analogous to the M-GP5 heterodimers of the *Arteriviridae* (13, 15, 53). For coronaviruses, the C-terminal domain of the N protein is critical for binding to the genomic RNA packaging signal. The N-terminal domain interacts with the endodomain of M to form virus particles (54–57). However, for PRRSV, the domain of the N protein that interacts with GP5 or nsp2 needs to be further explored. Our next goal is to identify which domain of nsp2 is important for its interaction with distinct viral envelope proteins. We speculate that this may depend on its different domains. In the future, with the development of high-resolution microscopy techniques, we may have detailed information on PRRSV assembly by deeply investigating its viral particles.

**Fig 12:**
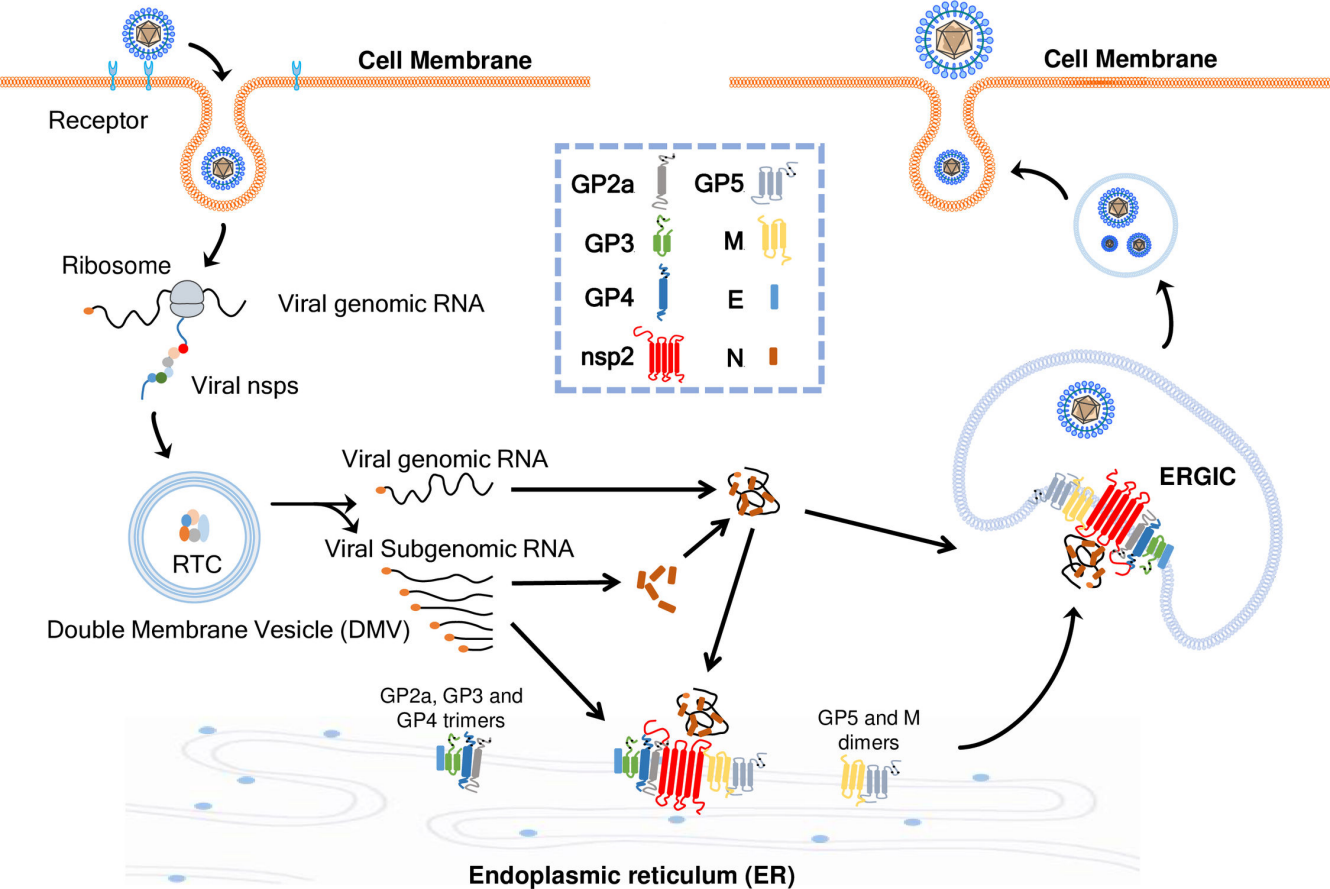
A proposed working model for HP-PRRSV assembly. HP-PRRSV enters target cells through receptor interactions. Subsequently, the viral genomic RNA is released, which encodes nonstructural proteins (nsps) that establish the replication-transcription complex (RTC) within double-membrane vesicles (DMVs), thereby facilitating the synthesis of both viral genomic RNA and a series of subgenomic RNAs. The viral RNA then directs the production of various viral proteins. Following protein expression, GP2a, GP3, and GP4 form a heterotrimer, while GP5 and M protein form a heterodimer. Viral full-length nsp2 promotes the assembly of N protein in conjunction with these structural proteins at the ER and ERGIC. Ultimately, mature virus particles are secreted.

In our current study, we found that PRRSV N protein assembles with various viral envelopes, mainly occurring at the ER and ERGIC. For SARS-CoV-2 assembly, the N protein is expressed first and adheres to folded ER membranes before viral assembly at the Golgi/ERGIC (58). We speculated that PRRSV N protein also attaches first to the ER or ERGIC cellular membranes, similar to coronaviruses. The location of nsp2 at the ER or ERGIC may have a function in concentrating viral structural proteins at these sites to facilitate viral particle assembly. In our study, we found limited co-localization of the TGN with the assembly complex (N protein, nsp2, and distinct viral envelope proteins). We believe this situation may be similar to that of coronaviruses; the envelope proteins, especially glycosylated proteins, need to be glycosylated in the Golgi apparatus and then returned to the ERGIC to assemble with N protein to complete the assembly process (59). Glycosylated structural proteins may travel by themselves or together from the Golgi apparatus to the ER or ERGIC to be incorporated into the nascent particle through an unknown mechanism. The sequences and motifs responsible for clustering in the ER or ERGIC within these proteins require further investigation. In this current study, we propose a working model illustrating the assembly of HP-PRRSV and the promotion of viral N protein assembly with viral envelope proteins by nsp2, as depicted in Fig. 12.

Our study also has some limitations as follows. First, we were unable to express and purify the full-length nsp2 and viral envelope proteins *in vitro*. This prevented us from conducting an *in vitro* assembly study. Secondly, due to our inability to generate antibodies against GP3 and GP4, we were unable to detect their interaction with nsp2 in our infection assay (Fig. 7). However, it is known that GP2a, GP3, and GP4 can form a complex. Therefore, if nsp2 mediates the assembly between N protein and GP2a, it is likely that the entire complex of GP2a-GP3-GP4 will also assemble with N protein. At last, most of our antibodies only reacted well with HP-PRRSV, whether other types of PRRSV also use full-length nsp2 to promote viral assembly needs further exploration.

Taken together, this study elucidates a novel functional role for nsp2, thereby providing crucial insights into HP-PRRSV assembly and enhancing our understanding of the lifecycle of these viruses. Consequently, these findings may offer an alternative approach to designing innovative antiviral or vaccine strategies (60).

## MATERIALS AND METHODS

### Cells, viruses, and antibodies

HEK293T, HeLa, and MARC-145 cells were cultured in Dulbecco’s modified Eagle’s medium (Gibco, USA) supplemented with 10% fetal bovine serum (FBS, Excell, FND500) at 37°C with 5% CO_2_. The PRRSV strain HuN4 was rescued from the PRRSV infectious clone (PRRSV HuN4-F5) (61–63). And a field-isolated HP-PRRSV-like strain (GenBank accession no. PQ178809) was stored in our lab. The mouse MAbs specific to PRRSV nsp2, N, and M were prepared as previously described (64, 65). Polyclonal antibodies against GP2a and GP5 were generated by immunizing mice with lipid-nanoparticle-encapsulated GP2a and Gp5-mRNA (mRNA-LNP) (Y. Tang, unpublished data).

### Plasmids

The pCAGGS-GP2a-Flag, pCAGGS-GP3-Flag, pCAGGS-GP4-Flag, pCAGGS-GP5-Flag, pCAGGS-M-Flag, pCAGGS-E-Flag, pCAGGS-N-Flag, pCAGGS-nsp2, and pCAGGS-N-HA plasmids were constructed in this study. The pVC-GP2a, pVC-GP3, pVC-GP4, pVC-GP5, pVC-M, pVC-E, pVC-N, pVN-N, and pVN-nsp2 plasmids were constructed by cloning GP2a, GP3, GP4, GP5, M, E, N, or nsp2 into the pVC or pVN vector (66). The pCAGGS-nsp2TF and pCAGGS-nsp2N were constructed by cloning the encoding area of nsp2TF or nsp2N into the PCAGGS empty vector, as Li et al. described (27, 46). The CNX-mcherry, TGN-mcherry, and EGRIC53-mCherry plasmids were synthesized by Sango Biotech. PCR primers are provided in the Supplementary data.

### Transfection and western blotting

The procedure was as per our previous reports (66–70). In brief, cells were seeded onto cell plates and transiently transfected with the indicated plasmid using X-tremeGENE HP DNA transfection Reagent (Roche, Switzerland). After 24 hours, the cells were lysed and subsequently subjected to western blot analysis.

### Co-immunoprecipitation assay

HEK293T cells were grown to approximately 70%–80% confluence in 6-well plates. The cells were then cotransfected with indicated plasmids or control plasmids. At 24 hpt, the cells were washed and lysed with RIPA lysis buffer containing PMSF for 30 minutes on ice. The cell lysate was collected and centrifuged at 12,000 × *g* for 10 minutes. The 100 µL of supernatant was taken as the input sample and mixed with 5× SDS-PAGE loading buffer then boiled at 100°C for 10 minutes. The remaining supernatant was added with 25 µL of FLAG M2 beads for IP assay (Sigma, USA), followed by western blotting.

The interactions between nsp2 and M, GP2a, GP5 or N during viral infection were conducted as follows. MARC-145 cells or PAMs were seeded in 6-well plates and infected with PRRSV HuN4 (MOI = 0.1) or HP-PRRSV-like strain when they reached 95% confluence. At 36–48 hours post-infection, whole lysates of PRRSV-infected cells were harvested and centrifuged. A volume of 100 µL supernatant was collected as the input sample, while the remaining supernatant was utilized for IP using Protein A/G Magnetic Beads (MCE, USA). The IP assay was initially performed employing an nsp2 antibody or N protein antibody.

### Bimolecular fluorescence complementation assay

The HEK293T cells were cultured in 12-well plates until they reached approximately 70% to 80% confluence. Subsequently, the cells were cotransfected with the indicated plasmids or control plasmids. After a transfection period of 24 hours, YFP fluorescence signals were directly observed using the LSM 980 Zeiss confocal microscope. Furthermore, at 24 hpt, the cells were washed once with PBS and treated with trypsin. Subsequently, the cells were collected and washed twice with PBS before detecting YFP fluorescence signals via flow cytometry.

### Immunofluorescence assay

The cells were seeded on 24-well plates with cell climbing sheets and cotransfected with the indicated plasmids using X-tremeGENE HP DNA transfection Reagent. After 24 hours of transfection, the cells were fixed, permeabilized, and blocked. Subsequently, specific primary antibodies were incubated with the cells at 37°C for 2 hours followed by three washes with PBS. Finally, appropriate secondary antibodies were applied to the cells before microscopic observation (68, 70).

### Statistical analysis

Statistical analysis was conducted using GraphPad Prism 8.0 software, and t-tests were used to determine statistical significance. A *P*-value of less than 0.05 was considered statistically significant.

## Data Availability

All data pertinent to this work are contained in this article or available upon request. For requests, please contact Yan-Dong Tang (tangyandong2008@163.com).

## SUPPLEMENTAL MATERIAL

Supplemental material jvi.01821-24-s0001.docx Fig. S1 to S4; Table S1.

ASM does not own the copyrights to Supplemental Material that may be linked to, or accessed through, an article. The authors have granted ASM a non-exclusive, world-wide license to publish the Supplemental Material files. Please contact the corresponding author directly for reuse.
