# Supplementary material for: The full-length nsp2 replicase contributes to viral assembly in highly pathogenic PRRSV-2
Source: J Virol. 2024 Nov 27;99(1):e01821-24. doi: 10.1128/jvi.01821-24 (PMC11784222; doi:10.1128/jvi.01821-24)
Supplement: Supplemental material — Fig. S1 to S4; Table S1. [file jvi.01821-24-s0001.docx]

**
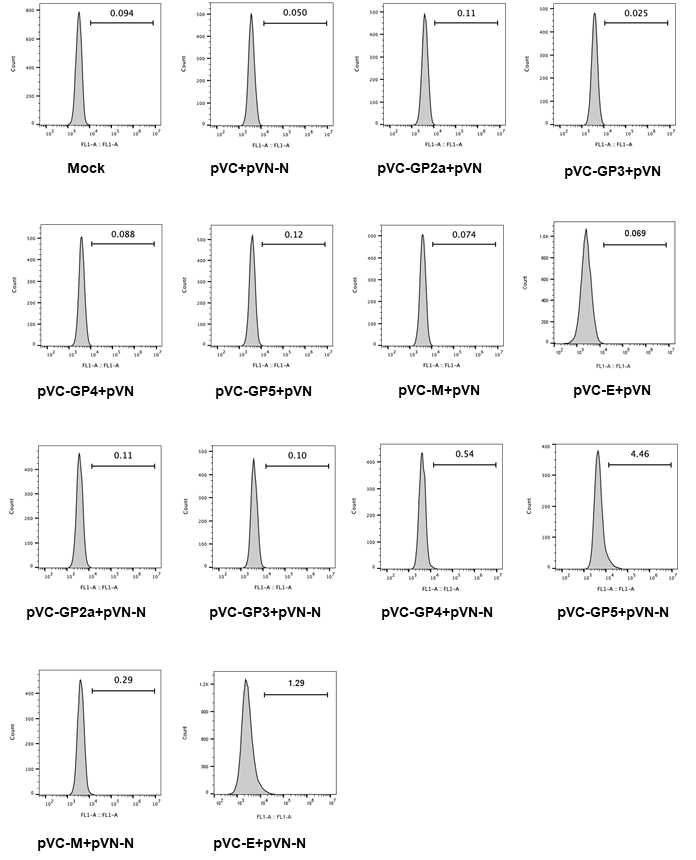
**

**Figure S1.** **Detection the potential interaction between the N protein and viral envelope proteins.** HEK293T cells were cotransfected pVN-N with pVC-GP2, pVC-GP3, pVC-GP4, pVC-GP5, pVC-M, pVC-E or control plasmids pVC. Cells were also transfected pVN with pVC-GP2, pVC-GP3, pVC-GP4, pVC-GP5, pVC-E or pVC-M as a control. At 24 hpt, the ratio of YFP fluorescence positive cells was detected via flow cytometry.

**
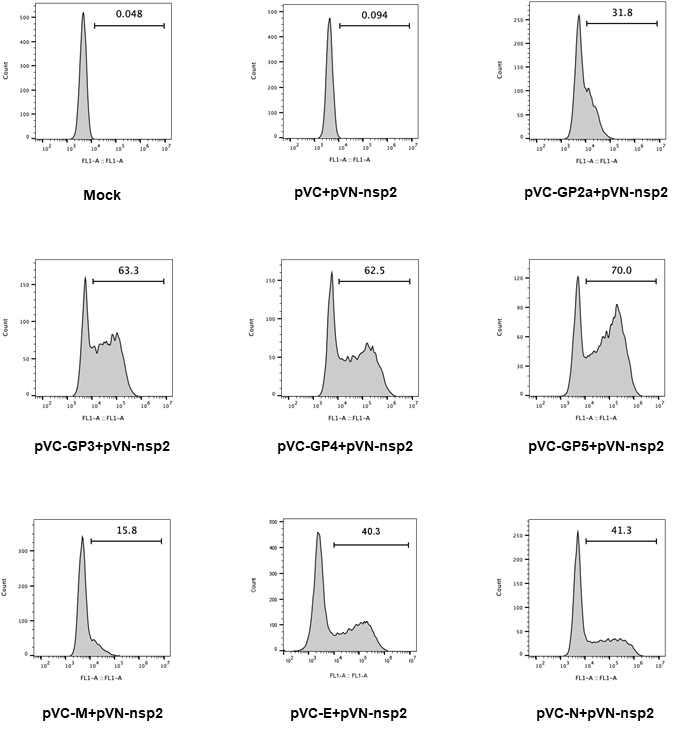
**

**Figure S2. Detection the potential interaction between the nsp2 and viral envelope proteins.** HEK293T cells were cotransfected pVN-nsp2 with pVC-GP2, pVC-GP3, pVC-GP4, pVC-GP5, pVC-M, pVC-E, pVC-N, or control plasmids pVC. At 24 hpt, the ratio of YFP fluorescence positive cells was detected via flow cytometry.

**
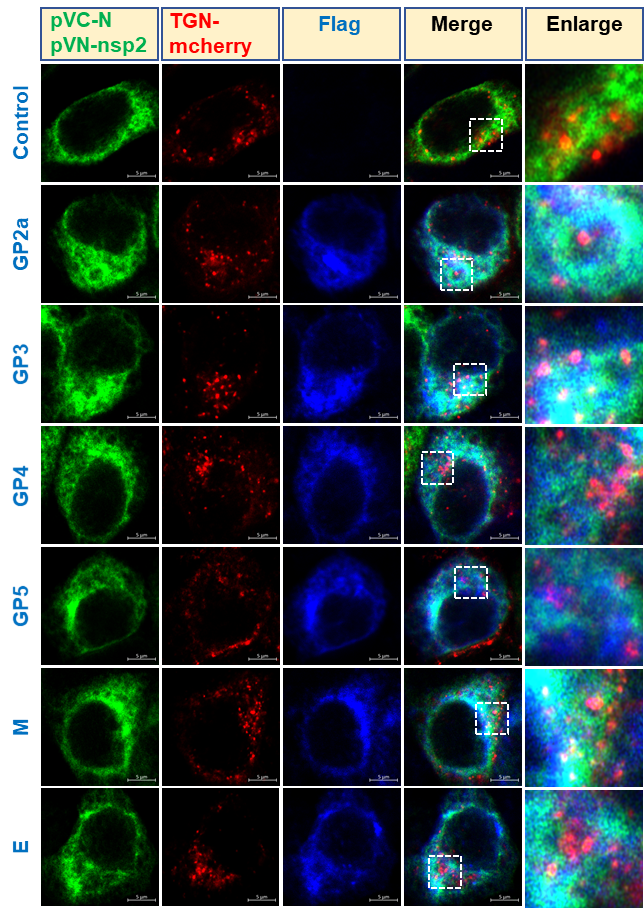
**

**Figure S3.** **Porcine arterivirus assembly not occurs in the TGN.** HeLa cells were cotransfected pVC-N and pVN-nsp2 with PRRSV GPs plasmid and TGN-mcherry. At 24 hpt, the cells were fixed, permeabilized, and immunostained. Fluorescent signals were observed using the LSM 980 Zeiss confocal microscope. Scale bars, 5 μm. The experiments were independently repeated three times, and representative data are shown.


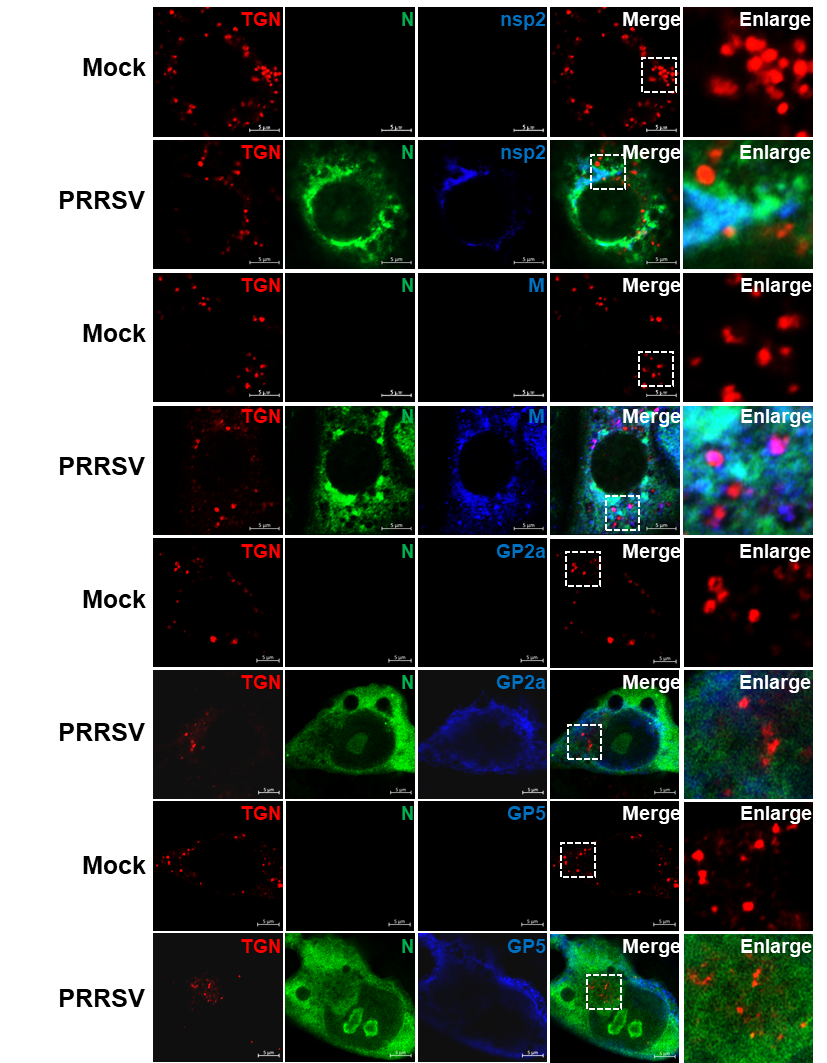


**Figure S4. The assembly site during PRRSV infection.** Marc-145 cells were transfected with TGN-mCherry. At 24 hpt, the cells were infected with PRRSV HuN4 (0.1 MOI), and uninfected cells were used as a control. At 48 hpi, the cells were fixed, permeabilized, and immunostained for PRRSV nsp2 or M protein (blue). Then, the cells were re-immunostained with the FITC-conjugated PRRSV/NP Mab (green). Scale bars, 5 μm. The experiments were independently repeated three times, and representative data are shown.

Table S1. Primers used in this study.

| Primers | Nucleotide sequence (5’-3’) |
| --- | --- |
| GP2a-Flag-F | CTGGCGTGTGACCGGCGGCTCTAGAATGAAATGGGGTCTA |
| GP2a-Flag-R | TTAAGATCTGCTAGCTCGAGTCACTTGTCGTCATCGTCTTTGTAGTCCCATGAGTTCAAAAG |
| GP3-Flag-F | CTGGCGTGTGACCGGCGGCTCTAGAATGGCTAATAGCTGT |
| GP3-Flag-R | TTAAGATCTGCTAGCTCGAGTCACTTGTCGTCATCGTCTTTGTAGTCTCGCCGTGCGGCACT |
| GP4-Flag-F | CTGGCGTGTGACCGGCGGCTCTAGAATGGCTGCGTCCTTT |
| GP4-Flag-R | TTAAGATCTGCTAGCTCGAGTCACTTGTCGTCATCGTCTTTGTAGTCAATTGCCAGTAGGAT |
| GP5-Flag-F | CTGGCGTGTGACCGGCGGCTCTAGAATGTTGGGGAAGTGC |
| GP5-Flag-R | TTAAGATCTGCTAGCTCGAGTCACTTGTCGTCATCGTCTTTGTAGTCGAGACGACCCCATTG |
| M-Flag-F | CTGGCGTGTGACCGGCGGCTCTAGAATGGGGTCGTCTCTA |
| M-Flag-R | TTAAGATCTGCTAGCTCGAGTCACTTGTCGTCATCGTCTTTGTAGTCTTTGGCATATTTAAC |
| N-Flag-F | CTGGCGTGTGACCGGCGGCTCTAGAATGCCAAATAACAAC |
| N-Flag-R | TTAAGATCTGCTAGCTCGAGTCACTTGTCGTCATCGTCTTTGTAGTCTGCTGAGGGTGATGC |
| nsp2-HA-F | CGATGTTCCAGATTACGCTGAATTCGGTGCCGGAAAGAGA |
| nsp2-HA-R | TAGTTAATTAAGATCTGCTAGCTCGAGTTACCCTGAAGGCTTGGA |
| nsp2TF-1-F | ACGATGTTCCAGATTACGCTGAATTCGGTGCCGGAAAGAGAGCAAG |
| nsp2TF-1-R | ACGAGGCTAAAAAAACCTGGCAGCTCAGTT |
| nsp2TF-2-F | AGGTTTTTTTAGCCTCGTTTCCCATCTCCC |
| nsp2TF-2-R | AGTTAATTAAGATCTGCTAGCTCGAGTTATTACACAAGATCCCCAGCAC |
| nsp2N-F | CGATGTTCCAGATTACGCTGAATTCGGTGCCGGAAAGAGAGCAAG |
| nsp2N-R | TTAATTAAGATCTGCTAGCTCGAGTTAAAAAACCTGGCAGCTCAGT |
| N-HA-F | CGATGTTCCAGATTACGCTGAATTCCCAAATAACAACGGC |
| N-HA-R | TAGTTAATTAAGATCTGCTAGCTCGAGTTATGCTGAGGGTGATGC |
| pVC-F | GGTGGAGGTGGAAGTGCCGA |
| pVC-R | CATCTCGAGAAGCTTAACTA |
| pVN-F | TCCGGAGTGAGCAAGGGCGA |
| pVN-R | CATCTCGAGAAGCTTAACTA |
| pVC-GP2a-F | TAGTTAAGCTTCTCGAGATGAAATGGGGTCTATGCAAAGC |
| pVC-GP2a-R | TCGGCACTTCCACCTCCACCCCATGAGTTCAAAAGAAAAG |
| pVC-GP3-F | TAGTTAAGCTTCTCGAGATGGCTAATAGCTGTACATTCCT |
| pVC-GP3-R | TCGGCACTTCCACCTCCACCTCGCCGTGCGGCACTGAGAA |
| pVC-GP4-F | TAGTTAAGCTTCTCGAGATGGCTGCGTCCTTTCTTTTCCT |
| pVC-GP4-R | TCGGCACTTCCACCTCCACCAATTGCCAGTAGGATGGCAA |
| pVC-GP5-F | TAGTTAAGCTTCTCGAGATGTTGGGGAAGTGCTTGACCGC |
| pVC-GP5-R | TCGGCACTTCCACCTCCACCGAGACGACCCCATTGTTCCG |
| pVC-M-F | TAGTTAAGCTTCTCGAGATGGGGTCGTCTCTAGACGACTT |
| pVC-M-R | TCGGCACTTCCACCTCCACCTTTGGCATATTTAACAAGGT |
| pVC-N-F | TAGTTAAGCTTCTCGAGATGCCAAATAACAACGGCAAGCA |
| pVC-N-R | TCGGCACTTCCACCTCCACCTGCTGAGGGTGATGCTGTGG |
| pVN-nsp2-F | TAGTTAAGCTTCTCGAGATGGGTGCCGGAAAGAGAGCAAG |
| pVN-nsp2-R | TCGCCCTTGCTCACTCCGGACCCTGAAGGCTTGGAAATTT |
| pVN-N-F | TAGTTAAGCTTCTCGAGATGCCAAATAACAACGGCAAGCA |
| pVN-N-R | TCGCCCTTGCTCACTCCGGATGCTGAGGGTGATGCTGTGG |
